# Notes from Past Meetings of the Homœopathic Medical Council*The Medical Council is a society composed of physicians practicing Homœopathy in Philadelphia and adjoining counties in Pennsylvania and New Jersey.—Eds.

**Published:** 1891-06

**Authors:** 


### NOTES FROM PAST MEETINGS OF THE HOMŒOPATHIC MEDICAL COUNCIL.*

Tuberculinum is indicated where the patient shows a constant disposition to catch cold. Catches cold, but does not know how.

Sabadilla, sensation in left testicle, as if it were revolving.

---

* The Medical Council is a society composed of physicians practicing Homœopathy in Philadelphia and adjoining counties in Pennsylvania and New Jersey.—EDS.

Alumina has enlargement of the right testicle.

Aurum, enlargement of left testicle.

Æthusa, headache goes off with a loose stool.

Silicea has headache, which comes on from exhausting, or hard work of any kind, whether mental or physical.

Medorrhinum is an excellent remedy for headache of exhaustion, or from hard work.

Dr. Mahlon Preston cured a case of gonorrhœa which had the indication, sore spot in the urethra, which commences with the erection, and continues until the erection ceases. The pain is as if torn. Alumina was the remedy.

Calc-carb. is indicated in gonorrhœa, when there is tickling at the meatus urinarius on urination.

Petroselinum is indicated where there is tickling, itching at the meatus urinarius on urinating.

Ratanhia is indicated in straining after stool, with intense pain and prolapsus of rectum.

Grindelia-robusta, Ammon-carb., and Carbo-veg. all have the indication, the patient sleeps into an aggravation. As soon as he begins to lose consciousness in sleep aggravation begins.

Dr. Robert Farley said that Grindelia-robusta has the symptom, upon going to sleep wakens up with shortness of breath. Respiration seems to stop. The following remedies all have the same symptom, sensation of smothering, or as if respiration ceased on falling asleep: Amm-c., Ant-t., Badiaga, Carb-an., Carb-veg., Dig., Graph., Grind-r., Lach., Op., Ran-b.

Lachesis is pre-eminent in all cases where the patient sleeps into an aggravation. As soon as he falls asleep aggravation sets in, waking him up.

Thus a baby when put to sleep would sleep twenty or thirty seconds, then waken up with a start and a scream, then fall to sleep again, when the same phenomena would be repeated. Lachesis$^{4m}$ was given, and was followed by an immediate cure.

Dr. Farley had a case of a child with catarrhal laryngitis. He gave several remedies without effect, until he noticed that as soon as she went to sleep she began to cough. He gave Lachesis, and the result was the patient was cured.

Phos., Lach., Anacard. are all better after eating. Aggravation sets in as soon as the stomach is empty, and relief occurs on eating.

