# Correction

**Published:** 1891-08

**Authors:** 


Correction:—Dr. J. B. Bell has written us that there is an error in the indications given in the first two lines on page 250, June number. It should read: Alumina, enlargement of *left* testicle. Aurum, enlargement of *right* testicle.

Dr. L. D. Rogers and Dr. Ida Wright Rogers, editors of the *People's Health Journal*, of Chicago, were attendants upon the International Homœopathic Congress lately assembled at Atlantic City.

Dr. L. D. Rogers is one of the leading homœopathic physicians who has just been appointed Professor of Surgery in the new German-American Homœopathic Medical College, established at Chicago, which is to be opened September 1st.

Dr. Prosper Bender has removed his office to No. 314 Boylston Street, opposite Arlington Street.

During July and August Dr. Bender will be at the Atlantic House, Nantasket, Mass., visiting the city Tuesdays, Thursdays, and Saturdays.

Office hours: 9 to 10 A. M., 2 to 4 P. M.

Boston, June 27th, 1891.

